# Fungal Pigments and Their Prospects in Different Industries

**DOI:** 10.3390/microorganisms7120604

**Published:** 2019-11-22

**Authors:** Ajay C. Lagashetti, Laurent Dufossé, Sanjay K. Singh, Paras N. Singh

**Affiliations:** 1 Biodiversity and Palaeobiology Group, National Fungal Culture Collection of India (NFCCI), MACS’ Agharkar Research Institute, G.G. Agarkar Road, Pune 411004, India; lagashettiajay@gmail.com (A.C.L.); pnsingh@aripune.org (P.N.S.); 2 Chimie et Biotechnologie des Produits Naturels & ESIROI Agroalimentaire, Université de la Réunion, 15 Avenue René Cassin, CS 92003, F-97744 Saint-Denis CEDEX, France

**Keywords:** color, natural pigments, fungal pigments, dyeing, textile fabrics

## Abstract

The public’s demand for natural, eco-friendly, and safe pigments is significantly increasing in the current era. Natural pigments, especially fungal pigments, are receiving more attention and seem to be in high demand worldwide. The immense advantages of fungal pigments over other natural or synthetic pigments have opened new avenues in the market for a wide range of applications in different industries. In addition to coloring properties, other beneficial attributes of fungal pigments, such as antimicrobial, anticancer, antioxidant, and cytotoxic activity, have expanded their use in different sectors. This review deals with the study of fungal pigments and their applications and sheds light on future prospects and challenges in the field of fungal pigments. Furthermore, the possible application of fungal pigments in the textile industry is also addressed.

## 1. Introduction

Color has always played an important role in the life of all organisms on Earth. Human life has become truly “colorful” due to the use of colors in all its aspects, including clothes, food, and furniture. Much archaeological evidence has shown that the use of pigments as coloring agents has been practiced since ancient times [1]. Pigments, especially synthetic ones, have occupied the entire market due to their wide range of applications in different industries since their discovery in the 19th century. Different attributes such as low production costs, ease of production, and superior coloring properties have largely contributed to the establishment of synthetic pigments in the market. However, the use of synthetic colors has been found to be detrimental to human health and the environment because of their many adverse impacts [2,3,4,5,6,7]. Many disadvantages of synthetic pigments, such as poor degradation, longer persistence, potential to cause cancers/allergies, etc., have increased the demand for natural, organic, and eco-friendly pigments in the current era.

The global response, as well as the demand for eco-friendly natural pigments, has significantly increased in recent decades due to their advantages over hazardous synthetic pigments. They are used as colorants, color intensifiers, additives, antioxidants, etc., in many industries including the textile, pharmaceutical, cosmetic, painting, food, and beverage industries [1,8]. In recent years, fungi have emerged among the prominent, eco-friendly sources of natural pigments. Easy processing, fast growth in cheap media, and weather-independent growth make them an excellent alternative to natural pigments. The present review highlights the role of fungi as small factories in pigment production and their potential application in different industries, including the textile industry.

## 2. Natural Pigments

Natural pigments are naturally derived pigments synthesized mainly by plants, animals, and microbes [5,9]. Most of the natural pigments used for different purposes since ancient times are produced from plants, such as annatto, grapes, indigo, beetroot, turmeric, madder, saffron, etc. [10,11]. However, the process of pigment production from plants may not be a good option because of various problems, such as season dependency, loss of vulnerable plant species due to their extensive use, variations in color shades and intensity, expensive production, and issues related to stability and solubility [2]. 

Nowadays, microorganisms, including bacteria, fungi, and algae, have been shown to be an excellent alternative source of natural pigments. For the large-scale production of pigments, microorganisms are more suitable, due to a clear understanding of their cultural techniques, processing, and ease of handling. Natural pigments from microbes, especially from bacteria and fungi, have been reported worldwide by many researchers [1,10,12,13,14,15,16,17,18,19,20]. Many bacterial species have been reported to possess potential for pigment production [10,21,22,23], but their pathogenic nature as well as associated toxicity have blocked production and commercialization. This eventually opened a new avenue for producing pigments from fungi and for their various applications.

## 3. Fungal Pigments

Fungi have been shown to be a good and readily available alternative source of natural pigments [1,20,24,25,26]. Fungi have immense advantages over plants such as season-independent pigment production, easy and fast growth in a cheap culture medium, production of pigments with different color shades and of more stable, soluble pigments, and easy processing [10,27]. Fungi belonging to the *Monascaceae*, *Trichocomaceae*, *Nectriaceae*, *Hypocreaceae*, *Pleosporaceae*, *Cordycipitaceae*, *Xylariaceae*, *Chaetomiaceae*, *Sordariaceae*, *Chlorociboriaceae*, *Hyaloscyphaceae*, *Hymenochaetaceae*, *Polyporaceae*, *Ophiostomataceae*, *Tremellaceae*, *Herpotrichiellaceae*, and *Tuberaceae* families have been described as potent pigment producers [8,12,20,25,26,28,29,30,31,32,33,34,35,36,37,38,39,40,41,42,43,44,45] (Table 1). These fungi are known to synthesize a variety of pigments as secondary metabolites. They are prolific producers of pigments belonging to several chemical classes, such as carotenoids, melanins, azaphilones, flavins, phenazines, quinones, monascin, violacein, indigo, etc. [16,25,26,46,47,48,49] (Table 1). 

**Figure 1:**
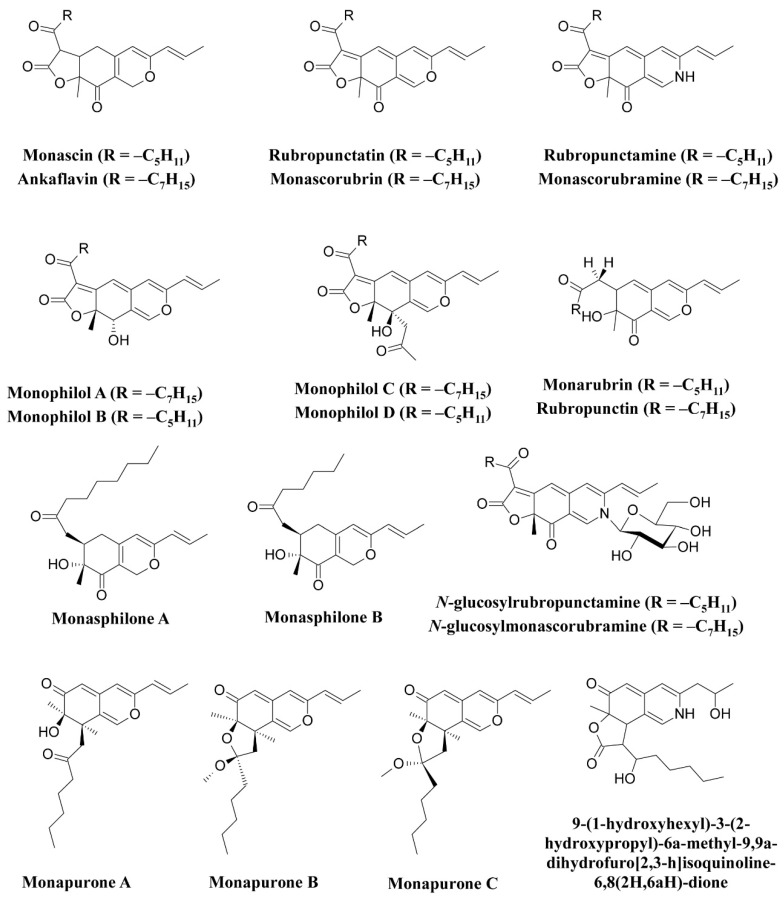
Pigments reported from *Monascus* species (*M. ruber* and *M. purpureus*), re-drawn from [52,54,56,57,58,59].

The use of *Monascus* pigments for the production of red mold rice (ang-kak) is the oldest recorded use of fungal pigments by humans. Certain species of *Monascus,* viz., *Monascus ruber* and *Monascus purpureus,* have been reported to be good potential producers of pigments worldwide. Studies have shown the potential of the red pigment produced by *M. ruber* as an important food colorant as well as food additive [50,51]. Many new pigments produced by *M. ruber*, such as *N*-glucosylrubropunctamine, *N*-glucosylmonascorubramine, monarubrin, rubropunctin, etc., have been discovered (Figure 1) [52,53,54]. Recently, researchers revealed the first detailed biosynthetic pathway of *Monascus* azophilone pigments (MonAzPs) in *M. ruber* M7, based on targeted gene knockouts, heterologous gene expression, as well as in vitro enzymatic and chemical reactions [55]. Along with *M. ruber*, *M. purpureus* was also reported to produce a variety of novel pigments, such as monapurone A–C, monasphilone A–B, monapilol A–D, and 9-(1-hydroxyhexyl)-3-(2-hydroxypropyl)-6a-methyl-9,9a-dihydrofuro[2,3-h] isoquinoline-6,8 (2H,6aH)-dione (Figure 1) [56,57,58,59]. Another study reports on the physicochemical (pH, light, and heat stability) properties of the red pigment of *M. purpureus* [60].

**Figure 2:**
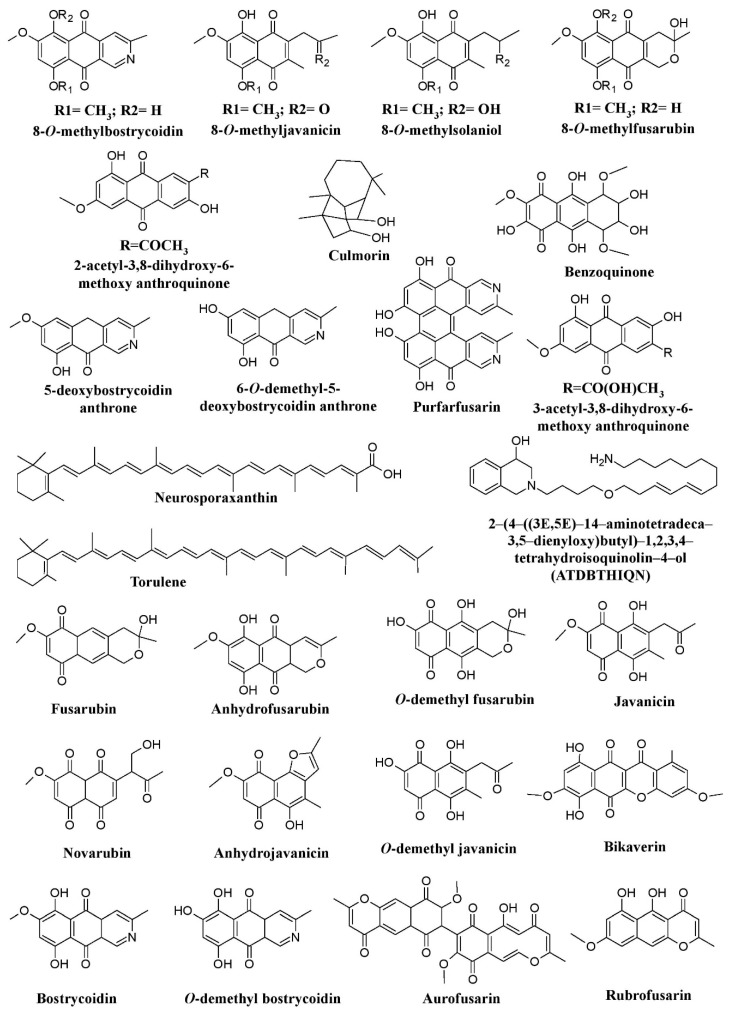
Pigments from fungal genera of Nectriaceae (*Fusarium*, *Fusicolla*, and *Albonectria*), re-drawn from [25,47,63,65,66,68].

Along with *Monascus*, many species of *Fusarium* have been reported for their capability to produce pigments. Studies have reported pigments such as bikaverin, nor-bikaverin, fusarubins, some naphthoquinone (8*-O*-methybostrycoidin, 8-*O*-methylfusarubin, 8-*O*-methylnectriafurone, 8-*O*-methyl-13-hydroxynorjavanicin, 8-*O*-methylanhydrofusarubinlactol, and 13-hydroxynorjavanicin), and a novel isoquinoline-type, pigment 2-(4-((3E,5E)-14-aminotetradeca-3,5-dienyloxy)butyl)-1,2,3,4-tetrahydroisoquinolin-4-ol (ATDBTHIQN), from *Fusarium fujikuroi* (formerly known as *Fusarium moniliforme*) (Figure 2) [25,63,65]. Similarly, differently colored naphthoquinones [bostrycoidin, 9-*O*-methylfusarubin, 5-*O*-methyljavanicin, 8-*O*-methylbostrycoidin, 1,4-naphthalenedione-3,8-dihydroxy-5,7-dimethoxy-2-(2-oxopropyl), 5-*O*-methylsolaniol, and 9-*O*-methylanhydrofusarubin], two anthraquinones compounds [2-acetyl-3,8-dihydroxy-6-methoxy anthraquinone and 2-(1-hydroxyethyl)-3,8-dihydroxy-6-methoxy anthraquinone], and polyketide pigment (bikaverin) were reported from *Fusarium oxysporum* (Figure 2) [25,47,64,67]. Another species of *Fusarium*, *Fusarium graminearum,* has been found to produce a variety of pigments such as 5-deoxybostrycoidin anthrone, 6-*O*-dimethyl- 5-deoxybostrycoidin anthrone, purpurfusarin, 6-*O*-demethyl-5-deoxybostrycoidin, 5-deoxybostrycoidin, and aurofusarin (Figure 2) [25,64,66,121]. 

A red pigment aurofusarin has been found to be produced by many species of Fusarium such as Fusarium culmorum, Fusarium sporotrichioides, Fusarim. acuminatum, Fusarium avenaceum, Fusarium poae, Fusarium crookwellens, Fusarium pseudograminearum, Fusarium sambucinum, and Fusarium tricinctum. Bikaverin has been reported to be produced by Fusarium lycopersici, and Fusarium vasinfectum. Fusarium solani and Fusarium verticillioides (currently known as F. fujikuroi) have been described to produce both aurofusarin and bikaverin (Figure 2) [25]. Similarly, benzoquinone has been reported from Fusarium sp. JN158 (Figure 2) [68]. A study has shown that the synthesis of major Fusarium carotenoids (neurosporaxanthin and β-carotene) is induced by light via transcriptional induction of the structural genes carRA, carB, carT, and carD [43]. Similarly, other members of the fungal family Nectriaceae, such as Albonectria rigidiuscula and Fusicolla aquaeductuum (formerly known as Fusarium decemcellulare and Fusarium aquaeductuum respectively) were reported for their pigment production potential (Figure 2) [43,64]. Recently, the biosynthetic pathway of chrysogine mediated by two-module non-ribosomal peptide synthetase (NRPS) gene cluster was discovered in Fusarium graminearum in which enhanced chrysogine production was observed upon overexpression of NRPS14 [122].

**Figure 3:**
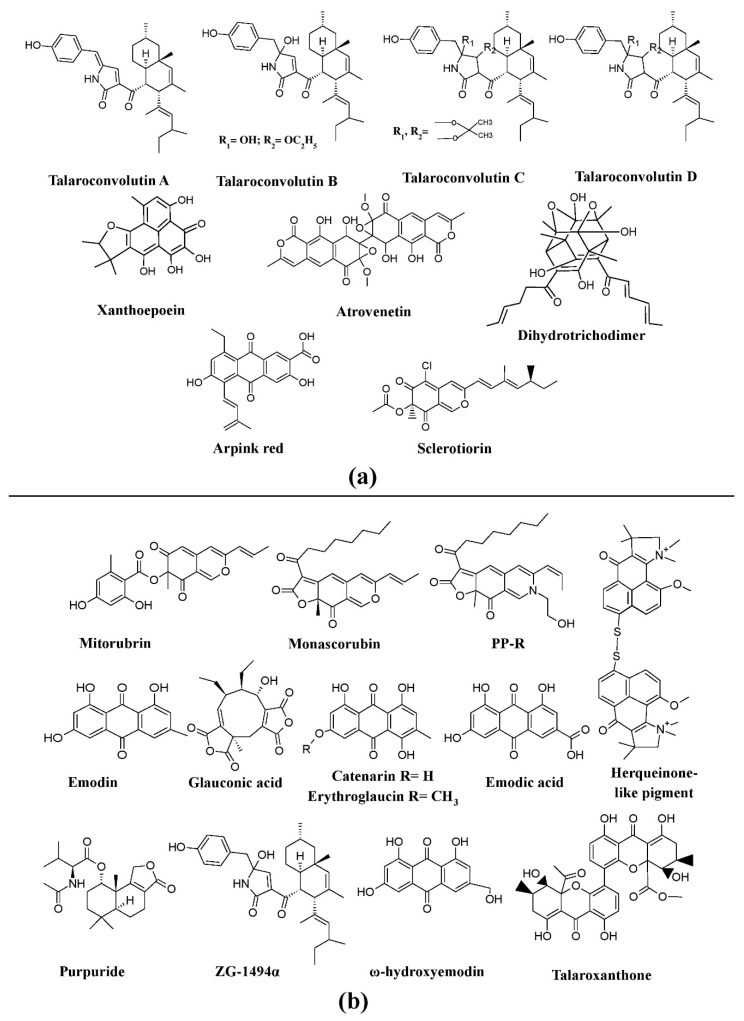
Pigments from the genera *Penicillium* and *Talaromyces*. (**a**) Different pigments produced by *Penicillium* species, re-drawn from [41,91,93,94,123]. (**b**) Various pigments produced by *Talaromyces* species, re-drawn from [100,101,107,109].

**Figure 4:**
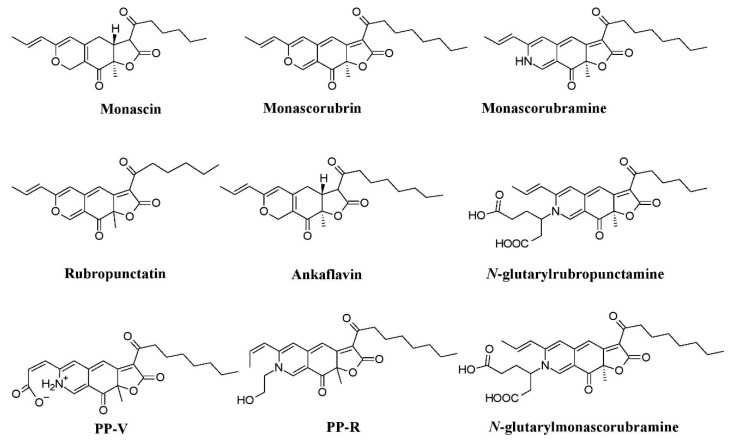
*Monascus*–like azaphilone pigments of *Penicillium* and *Talaromyces* species, re-drawn from [25,95,106].

Many investigations report *Penicillium* as potent producers of pigment [25,61,96,97,98], such as arpink red^TM^ (first commercial red colorant), talaroconvolutins A–D, sclerotiorin, xanthoepocin, atrovenetin, and dihydrotrichodimerol discovered from *Penicillum oxalicum* var. *armeniaca*, *Penicillum convolutum* (formerly known as *Talaromyces convolutes*), *Penicillum mallochii*, *Penicillum simplicissimum*, *Penicillum melinii*, and *Penicillum flavigenum*, respectively (Figure 3a) [41,91,93,94,123]. An uncharacterized red pigment has been reported from *Penicillium miczynskii* [71]. Besides, many other *Monascus*-like pigments such as PP-V [(10Z)-12-carboxylmonascorubramine] and PP-R [(10Z)-7-(2-hydroxyethyl)-monascorubramine] have been reported from *Penicillium* (Figure 4) [95]. A biosynthetic pathway for the yellow pigment chrysogine from *Penicillium chrysogenum* has been proposed recently [92]. 

*Talaromyces* spp. have been reported as a source of pigments by many researchers. The pigment production ability of *Talaromyces purpureogenus* (formerly known as *Penicillium purpureogenum*) was evaluated by many researchers [102,104,105]. Studies report the production of a herqueinone-like pigment from *Talaromyces marneffei* (formerly known as *Penicillium marneffei*), *Monascus*-like azaphilone pigments (*N*-glutarylmonascorubramine and *N*-glutarylrubropunctamine) from *Talaromyces purpureogenus* (formerly known as *Penicillium purpureogenum*), industrially important red pigments (mitorubrin, monascorubrin, PP-R, glauconic acid, purpuride, and ZG-1494α) from *Talaromyces atroroseus*, trihydroxyanthraquinones (emodin, erythroglaucin, and catenarin) from *Talaromyces stipitatus*, and a xanthone dimer (talaroxanthone) from *Talaromyces* sp. (Figure 3b) [100,101,103,107,109]. An uncharacterized red pigment was discovered from *Talaromyces siamensis* under submerged fermentation [71]. Moreover, other species of *Talaromyces, Talaromyces aculeatus*, *Talaromyces atroroseus*, *Talaromyces albobiverticillius*, *Talaromyces cnidii*, *Talaromyces coalescens*, *Talaromyces pinophilus*, *Talaromyces purpurogenus*, *Talaromyces funiculosus*, *Talaromyces amestolkiae*, *Talaromyces ruber*, *Talaromyces stollii*, and *Talaromyces verruculosus* have been reported to have the ability to produce *Monascus*-like azaphilone pigments (Figure 4) [25,106].

**Figure 5:**
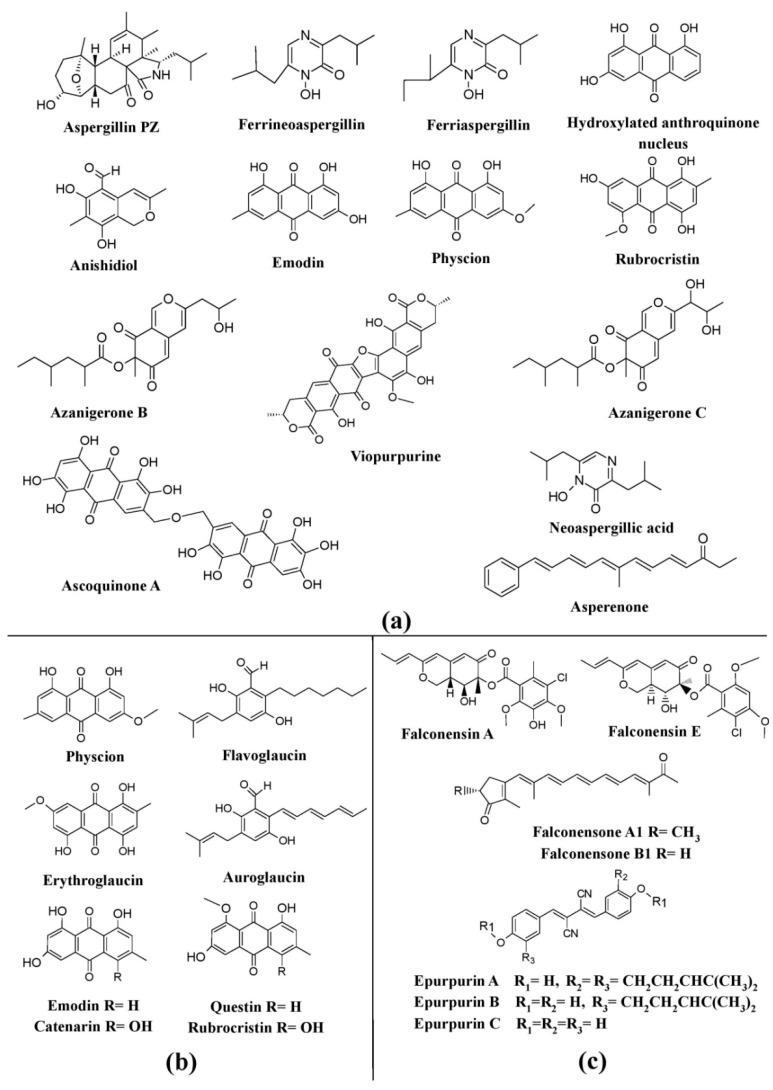
Pigments from the genus *Aspergillus* and its teleomorphic genera. (**a**) Structures of pigments produced by *Aspergillus* species. (**b**) Pigments produced by species of *Eurotium* (teleomorph of *Aspergillus*). (**c**) Pigments produced by species of *Emericella* (teleomorph of *Aspergillus*), re-drawn from [25].

Several members of the genus *Aspergillus*, such as *Aspergillus niger*, have been known to synthesize a wide variety of pigments, such as aspergillin, asperenone, azaphilones (azanigerones A–F), and melanin (Figure 5a) [25,110,114,115]. *Aspergillus nidulans* was reported to produce ascoquinone A, norsolorinic acid, and melanin [25,112,113], whereas *Aspergillus fumigatus* was reported to produce melanin and melanin-like pigments [25,111]. In addition, a variety of other pigments such as asperenone, anishidiol, neoaspergillic acid, sterigmatocystin, and an uncharacterized yellow pigment have been discovered from *Aspergillus nishimurae*, *Aspergillus awamori*, *Aspergillus sclerotiorum*, *Aspergillus versicolor*, and *Aspergillus terreus*, respectively [25,91,110,116,118]. Many other species of *Aspergillus* such as *Aspergillus glaucus*, *Aspergillus cristatus*, and *Aspergillus repens* have been reported to produce a variety of hydroxyanthraquinone pigments, emodin, physcion, questin, erythroglaucin, catenarin, and rubrocristin; while *Aspergillus melleus*, *Aspergillus ochraceus*, *Aspergillus sulphureus*, and *Aspergillus westerdijkiae* have been described to be major producers of polyketide-based pigments (rubrosulfin, viomellein, viopurpurin, and xanthomegnin) (Figure 5a) [25]. In addition to this, other pigments such as ferriaspergillin, ferrineoaspergillin, and an uncharacterized yellow pigment have also been reported from the genus *Aspergillus* (Figure 5a) [119,120].

Certain teleomorphic species of *Aspergillus* have been described as producers of a variety of pigments. Some of the well-known azaphilone pigments such as falconensins A–H, zeorin, falconensones A1 and B2 have been reported from *Emericella falconensis* and *Emericella fruticulosa* (currently known as *Aspergillus falconensis* and *Aspergillus fruticulosus*, respectively), epurpurins A-C from *Emericella purpurea* (currently known as *Aspergillus purpureus*), and the pigment sterigmatocystin from *Emericella rugulosus*, *Emericella parvathecia*, and *Emericella nidulans* (currently known as *Aspergillus rugulosus*, *Aspergillus parvathecia*, and *Aspergillus nidulans*) (Figure 5c). Similarly, other *Aspergillus* spp. such as *Aspergillus amstelodami*, *Aspergillus chevalieri*, *Aspergillus glaucus*, *Aspergillus umbrosus*, *Aspergillus spiculosus*, *Aspergillus glaber*, *Aspergillus echinulatum*, *Aspergillus tonophilus*, *Aspergillus intermedius*, *Aspergillus leucocarpus*, *Aspergillus ruber,* and *Aspergillus cristatus* (which were formerly known as *Eurotium amstelodami*, *Eurotium chevalieri*, *Eurotium herbariorum*, *Eurotium umbrosum*, *Eurotium spiculosum*, *Eurotium spiculosum*, *Eurotium echinulatum*, *Eurotium tonophilum*, *Eurotium intermedium*, *Eurotium leucocarpum*, *Eurotium rubrum*, and *Eurotium cristatum*, respectively) have also been reported to produce pigments such as physcion, erythroglaucin, flavoglaucin, auroglaucin, catenarin, rubrocristin, and emodin (Figure 5b) [25].

**Figure 6:**
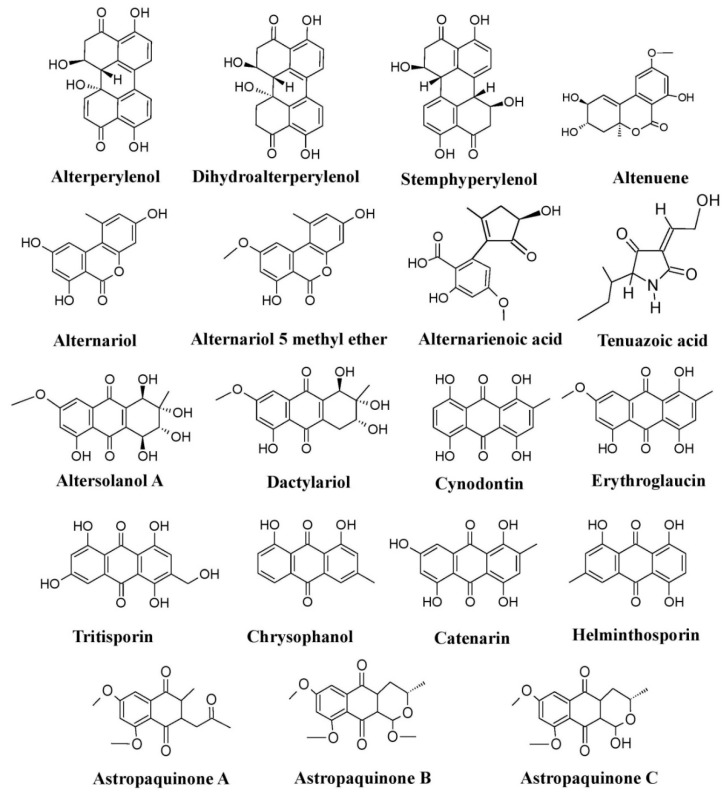
Pigments produced by members of the fungal family Pleosporaceae (species of *Alternaria*, *Curvularia*, *Astrosphaeriella*, and *Pyrenophora*), re-drawn from [25,76,77,78].

**Figure 7:**
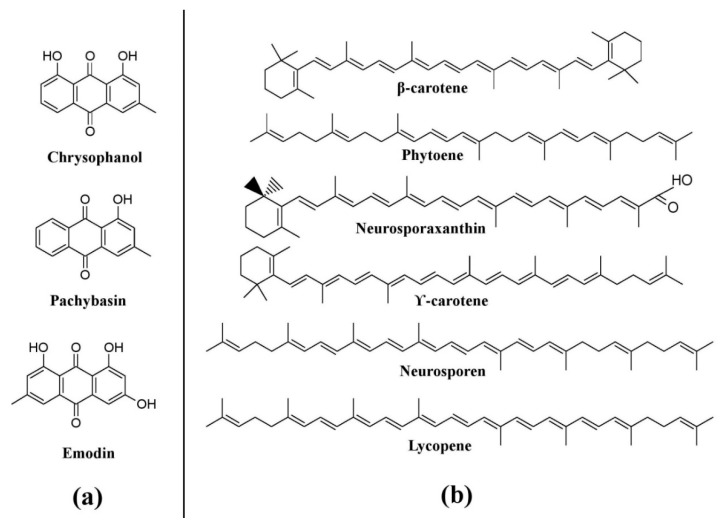
Pigments from other fungi. (**a**) Pigments from *Trichoderma* species, based on [25]. (**b**) Pigments from *Neurospora* species, re-drawn from [25,90].

Members of different genera of the fungal family Pleosporaceae (*Alternaria*, *Curvularia*, *Pyrenophora*, etc.) have immense potential for pigment production. Species of *Alternaria* such as *Alternaria alternata*, *Alternaria solani*, *Alternaria porri*, and *Alternaria tomatophila* have been reported to produce a variety of pigments such as dactylariol, alterperylenol, dihydroalterperylenol, alternariol, alternariol-5-methyl ether, altenuene, alternarienoic acid, tenuazoic acid, stemphyperylenol, and altersolanol A (Figure 6) [25,76,77,78]. Also, other members of the Pleosporaceae, *Curvularia* and *Pyrenophora*, have been known to produce different types of pigments, e.g., *Curvularia lunata* produces hydroxyanthraquinone pigments such as chrysophanol, cynodontin, helminthosporin, erythroglaucin, and catenarin, whereas different species of *Pyrenophora* such as *Pyrenophora teres*, *Pyrenophora graminea*, *Pyrenophora tritici-repentis*, *Pyrenophora grahamii*, *Pyrenophora dictyoides*, and *Pyrenophora chaetomioides* (which were previously known as *Drechslera teres*, *Drechslera graminea*, *Drechslera tritici-repentis*, *Drechslera phlei*, *Drechslera dictyoides*, *Drechslera avenae*, respectively) have also been reported to produce hydroxyanthraquinone pigments such as cynodontin, erythroglaucin, catenarin, helminthosporin, and tritisporin (Figure 6) [25,61]. *Trichoderma*, a well-known bio-control agent, has been known to produce a variety of pigments [25,124]. Several hydroxyanthraquinones such as pachybasin, chrysophanol, emodin, T22 azaphilone, 1-hydroxy-3-methyl-anthraquinone, 2,4,5,7-tetrahydroxyanthraquinone, 1,3,6,8-tetrahydroxyanthraquinone, and 1,8-dihydroxy-3-methyl-anthraquinone, have been reported from different species of *Trichoderma* (*Trichoderma harzianum*, *Trichoderma polysporum*, *Trichoderma viride*, and *Trichoderma aureoviride*) (Figure 7a) [25], whereas *Trichoderma afrharzianum*, *Trichoderma pyramidale*, and *Trichoderma* sp. 1 are reported to produce uncharacterized yellow pigments in submerged fermentation [71]. Studies have also revealed that certain species of *Neurospora*, such as *Neurospora crassa*, *Neurospora sitophila*, and *Neurospora intermedia* produce a variety of carotenoids such as phytoene, β-carotene, γ-carotene, lycopene, neurosporene, and neurosporaxanthin (Figure 7b) [25,26,90].

**Figure 8:**
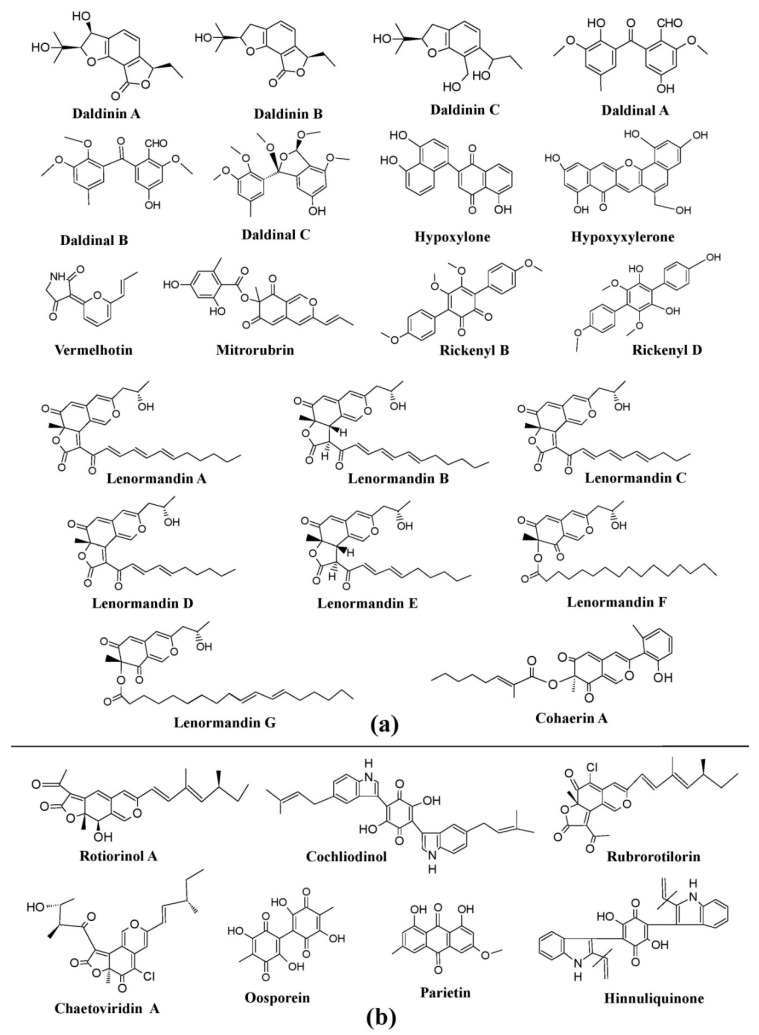
Pigments from the fungi of Xylariaceae and Chaetomiaceae families. (**a**) Pigments from members of the Xylariaceae family (species of *Daldinia*, *Hypoxylon*, and *Jackrogersella*), re-drawn from [25]. (**b**) Pigments from members of the Chaetomiaceae family (species of *Chaetomium* and *Achaetomium*) and Hypoxylaceae, re-drawn from [25,84].

Many genera of the Xylariaceae family, such as *Daldinia*, *Hypoxylon*, *Jackrogersella*, etc., have a great capability to synthesize pigments of very diverse colors and hues [25]. A variety of interesting pigments such as BNT (1,1ˊ-Binaphthalene-4,4ˊ-5,5́-tetrol), daldinol, daldinal A–C, and daldinin A–C have been reported from different species of *Daldinia*, such as *Daldinia bambusicola*, D*aldinia caldariorum*, *Daldinia concentrica*, *Daldinia eschscholzii*, *Daldinia childiae*, *Daldinia clavata*, *Daldinia fissa*, *Daldinia grandis*, *Daldinia lloydi*, *Daldinia loculata*, *Daldinia petriniae*, *Daldinia singularis* (Figure 8a). Similarly, several cohaerin variants (cohaerin A–K), multiformin A, and sassafrins D have been obtained from *Jackrogersella cohaerens* (formerly known as *Annulohypoxylon cohaerens*) (Figure 8a). Besides this, several species of *Hypoxylon* were declared to produce diverse pigments e.g., *Hypoxylon fragiforme* (hypoxyxylerone, cytochalasin H, fragiformins A–B, and mitorubrin), *Hypoxylon howeanum* (mitorubrin and azaphilones), *Hypoxylon lechatii* (vermelhotin and hypoxyvermelhotins A–C), *Hypoxylon fuscum* (daldinin A–C), *Hypoxylon fulvo-sulphureum* (mitorubrinol derivatives), *Hypoxylon sclerophaeum* (hypoxylone), *Hypoxylon rickii* (rickenyl B and D), *Hypoxylon lenormandii* and *Hypoxylon jaklitschii* (lenormandins A-G), *Hypoxylon rubiginosum* (mitorubrin, rubiginosin, and hypomiltin) (Figure 8a). Members of the Chaetomiaceae family also exhibit potential of pigment production. *Chaetomium cupreum* has been mentioned to produce red azaphilone pigments, oosporein, rotiorinols A–C, rubrorotiorin, whereas *Chaetomium globosum* produces yellow azaphilone pigments (chaetoviridins A–D), chaetoglobin A–B, chaetomugilins A–F, and cochliodinol (Figure 8b). Production of parietin (hydroxyanthraquinone pigment) has also been revealed from the *Achaetomium* sp. (Figure 8b) [25].

**Figure 9:**
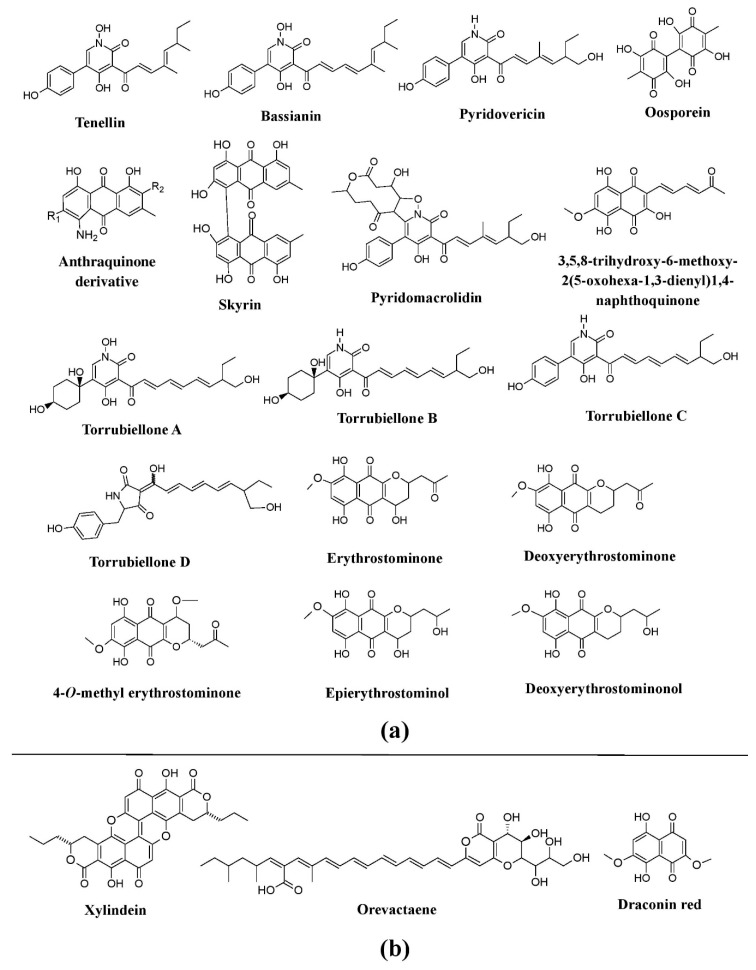
Pigments from the fungi of the Cordycipitaceae family and some other group. (**a**) Pigments from members of the families Cordycipitaceae (species of *Beauveria*, *Torrubiella*, *Cordyceps*, *Hyperdermium*, and *Lecanicillium*) and Ophiocordycipitaceae (*Ophiocordyceps* sp.), re-drawn from [25,41,73,74,75,125]. (**b**) Pigments known from other groups of fungi (species of *Chlorociboria*, *Scytalidium,* and *Epicoccum*), re-drawn from [37,41].

Also, the genera belonging to the family Cordycipitaceae such as *Torrubiella*, *Cordyceps*, *Beauveria*, *Hyperdermium*, and *Lecanicillium* have been revealed to be promising producers of bioactive pigments, e.g., tenellin and bassianin are reported from *Beauveria bassiana* and *Beauveria brongniartii* (formerly known as *Beauveria tenella*), pyridovericin and pyridomacrolidin from *Beauveria bassiana*, torrubiellones A–D from the genus *Torubiella*, oosporein from *Lecanicillium aphanocladii*, whereas anthraquinone-related compounds are reported from *Cordyceps farinosa* (formerly known as *Isaria farinosa*) (Figure 9a) [41,73,74,75,125]. Similarly, the pigments erythrostominone, 4-*O*-methyl erythrostominone, deoxyerythrostominone, deoxyerythrostominol, epierythrostominol, and 3,5,8-TMON (3,5,8-trihydroxy-6-methoxy-2-(5-oxohexa-1,3-dienyl)-1,4-naphthoquinone) have been reported from *Ophiocordyceps unilateralis* (formerly known as *Cordyceps unilateralis*), and skyrin from *Hyperdermium bertonii* (Figure 9a) [25].

**Figure 10:**
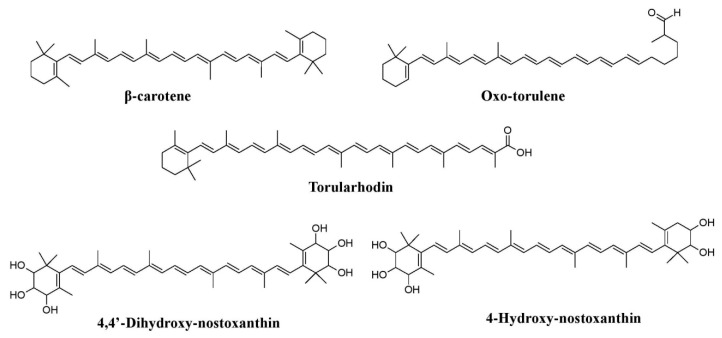
Pigments reported from yeasts such as *Rhodotorula glutini* and *Xanthophyllomyces dendrorhous*, re-drawn from [13,128].

Apart from this, studies have reported the production of the pigment xylindein from *Chlorociboria aeruginosa* and *Chlorociboria aeruginascens*, draconin red from *Scytalidium cuboideum*, and a yellow pigment from *Scytalidiium ganodermophthorum* and *Scytalidium lignicola*. Other pigments, such as orevactaene produced from *Epicoccum nigrum*, emodin, ω-hydroxyemodin, and emodic acid from *Hamigera avellanea* (formerly known as *Talaromyces avellaneus*) are also known (Figure 3b, Figure 9b) [33,36,37,39,41,109]. Recently, fungi such as *Sanghuangporus baumii* and *Clonostachys intermedia* have been found to produce a yellow pigment under submerged fermentation [71]. Production of melanin was reported from different groups of fungi such as *Phyllosticta capitalensis*, *Xylaria polymorpha*, *Trametes versicolor*, *Inonotus hispidus*, *Oxyporus populinus*, *Fomes fomentarius*, *Exophiala dermatitidis, Tuber melanosporum, Sporothrix schenckii*, and *Cryptococcus neoformans* [29,34,35,44,80,81,83]. Similarly, a study has shown the possible industrial application of the red pigment produced by *Paecilomyces sinclairii* [126]. Besides filamentous fungi, certain genera of yeasts (*Rhodotorula*, *Sporidiobolus*, *Sporobolomyces* and *Xanthophyllomyces*) have also been known as pigment producers. Different species of *Rhodotorula (Rhodotorula glutinis*, *Rhodotorula mucilaginosa (syn. Rhodotorula rubra)*, *Rhodotorula babjevae*, *Rhodotorula toruloides Rhodotorula graminis*), *Sporidiobolus (Sporidiobolus pararoseus*, *Sporidiobolus johnsonii)*, and *Sporobolomyces* (*Sporobolomyces uberrimus*, *Sporobolomyces salmonicolor*) have been reported to be prolific producers of torulin and torularhodin [127]. Researchers have discovered pigments such as β-carotene, torulene, and torularhodin from *Rhodotorula glutini* and multi-hydroxy carotenoids (4,4′-dihydroxy-nostoxanthin and 4-hydroxy-nostoxanthin) from *Xanthophyllomyces dendrorhous* (Figure 10) [13,128].

**Figure 11:**
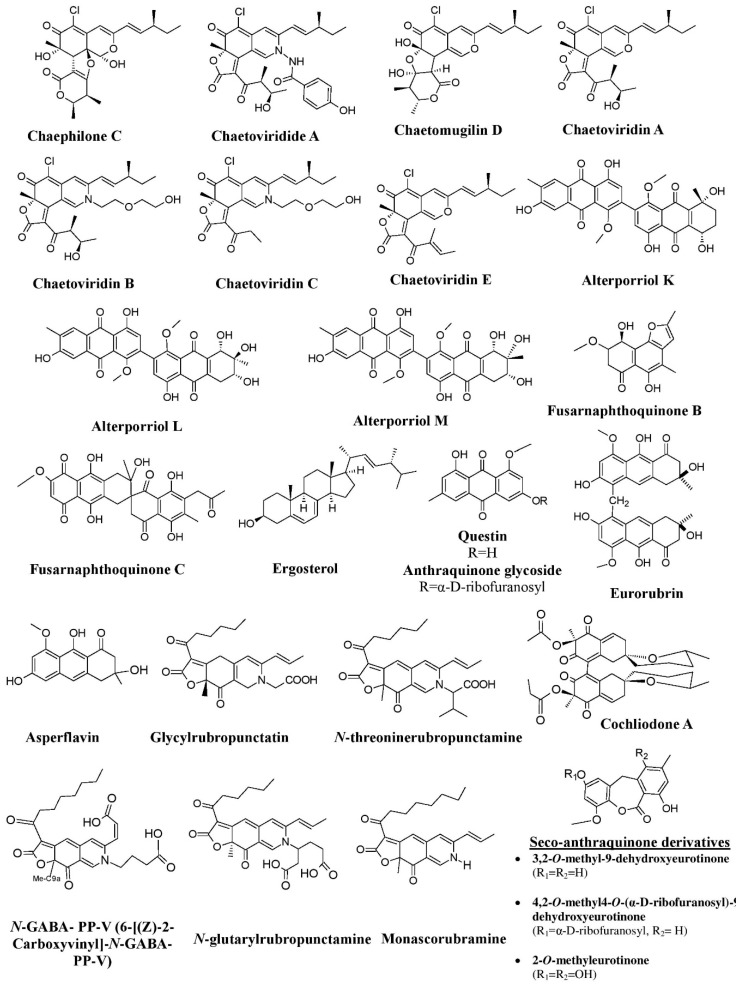
Pigments produced by marine fungal isolates, re-drawn from [69,72,79,82,117].

In addition to terrestrial fungi, marine fungi are also very good producers of a variety of unique pigments having promising therapeutic and industrial applications [129,130]. Studies on marine fungi by many researchers have reported a wide range of pigments and hues, e.g., a variety of anthraquinone pigments [asperflavin, 2-*O*-methyleurotinone, questin, eurorubrin, 2-*O*-methyl-9-dehydroxyeurotinone, 2-*O*-methyl- 4-*O*-(α-D-ribofuranosyl)-9-dehydroxyeurotinone, and 6, 3-*O*-(α-D-ribofuranosyl)-questin] from the mangrove endophytic fungus *A. ruber* (formerly known as *Eurotium rubrum*), fusarnaphthoquinones B and fusarnaphthoquinones C from the sea fan-derived fungi *Fusarium* species, and bianthraquinone derivatives (alterporriol K, alterporriol L, and alterporriol M) from mangrove endophytic *Alternaria* sp. (Figure 11) [69,79,117]. Researchers have also investigated the red pigment production from mangrove fungus *Penicillium* sp. and a yellow pigment production from the marine sponge-associated fungus *Trichoderma parareesei* [70,99].

Also, many studies have revealed the production of polyketide pigments (*N*-threonine rubropunctamine) and chlorinated azaphilone pigments (chaephilone-C, chaetoviridides-A, chaetoviridides-B, chaetoviridides-C) from marine fungal isolates of *Talaromyces* spp. and *Chaetomium* sp., respectively (Figure 11) [72,82]. A recent study has reported a novel pigment, *N*-GABA-PP-V (6-[(Z)-2-Carboxyvinyl]-*N*-GABA-PP-V), along with *N*-threonine-monascorubramine, *N*-glutaryl-rubropunctamine, and PP-O from the marine-derived fungus *Talaromyces albobiverticillius* (Figure 11) [131]. Many antarctic fungi have also been discovered to produces pigments of different chemical classes and characteristics. A number of yeast and filamentous fungi isolated from the different samples collected from Antarctic regions have been reported to produce a variety of pigments with different colors [86].

## 4. Optimization for Enhancement of Pigment Production

Most of the investigators have focused their study on the enhancement of pigment production from different fungal strains such as *Monascus*, *Penicillium*, *Talaromyces*, *Fusarium*, etc., by optimizing various fermentation parameters such as media, media composition, pH, temperature, light intensity, orbital speed, etc. [26,132,133,134,135]. Some studies have reported about the assessment of the pigment production potential of different fungi on natural substrates (rice, corn, wheat, cassava, whole sorghum grain, dehulled sorghum grain, and sorghum bran) and on different agro-industrial residues (feather meal, fish meal, cheese whey, grape waste, soybean protein, soybean meal, chicken feather and rice husk, orange processing waste) [134,136,137,138]. Enhancement in xylindein production was reported in *Chlorociboria aeruginascens* upon addition of test woods (*Acer saccharum*, *Populus tremuloides*, spalted *P. tremuloides*, and *Ailanthus altissima*) in agar-based media [33].

Some studies have also evaluated the effect of different sugar sources such as glucose, fructose, lactose, sucrose, and maltose on pigment production by the species of *Monascus*. Results of these studies have shown that maximum pigment production was acheived in media with fructose as a carbon source for *M. purpureus*, and lactose as a carbon source for *M. ruber* [132,139]. Studies have also discovered that the addition of different nitrogen sources such as ammonium, peptone, sodium nitrate, glutamic acid, monosodium glutamate, 6-furturylaminopurine, and tryptophan could enhance the yield of pigment, alter the hue of the fermentation liquid, and also improve light stability of the pigments of *Monascus* species [132,140,141,142,143]. NaCl has been proved to be a very good enhancer that stimulates pigment production and inhibits citrinin production in *M. purpureus* without affecting the growth of the fungus [144]. A study on the effect of nutrients on pigment production of *C. aeruginascens* shows that high biomass but no pigment production was observed in media with high nutrient concentration, whereas low biomass and high pigmentation was observed in media with low nitrogen concentration [145]. Investigators have also found variations in the yield, color characteristics (hue and chroma values), and structure of the pigments of *Monascus* species with respect to the type of amino acids in the media [146,147]. Beside this, the pH of the media also plays an important role in pigment production. In the case of *Monascus* species (*M. purpureus*, *M. major,* and *M. rubiginosus*), pH optimization studies have shown that a low pH of the media increases pigment production [140,146,148]. Another study has revealed that the pH of the substrate plays an important role in melanin production by *X. polymorpha*, *T. versicolor*, *Cerioporus squamosus* (formerly known as *Polyporus squamosus*), *Lentinus brumalis* (formerly known as *Polyporus brumalis*), *F. fomentarius* and *I. hispidus.* The maximum pigment production was observed in the pH range from 4.5 to 5.5 [35]. Similar studies in other fungi such as *Penicillium purpurogenum*, *P. aculeatum, A. niger*, *Altemaria* sp., *Fusarium* sp., *C. aeruginascens*, have shown that the optimum pH for maximum pigment production varies with the fungal species in submerged fermentation [35,149,150,151,152].

Along with chemical parameters, physical parameters such as temperature, light intensity, color of light, agitation speed, and oxygen supply have an impact on pigment production. Studies have also been reported showing the influence of temperature on the biosynthesis of pigments by certain fungal isolates such as *M. ruber*, *T. purpureogenus* (formerly known as *P. purpurogenum*), *C. aeruginascens*, etc. [150,152,153]. Enhancement of yellow pigment production in a *Monascus anka* mutant strain under submerged fermentation using a two-stage agitation speed control strategy (400 rpm followed by 300 rpm) has been successfuly reported [154]. A study has also revealed that a sufficient supply of oxygen is necessary for xylindein production by *C. aeruginascens* [152]. The impact of darkness and different color light on the yield of extracellular and intracellular pigment and biomass has been assessed by various investigators. Most of the studies have shown that incubation in total darkness resulted in enhanced biomass and pigment production [152,155,156]. Studies have also reported that there is an enhancement in the pigment production in the case of *A. alternata* and *M. ruber* when exposed to blue and red light, respectively [156,157], and in *F. oxysporum* when exposed to blue and green light [158]. In contrast, reduction in biomass and pigment yield has been observed in *I. farinosa*, *E. nidulans*, *F. verticillioides*, *P. purpurogenum* (currently known as *C. farinosa*, *A. nidulans*, *F. fujikuroi*, *T. purpureogenus*, respectively), and *M. purpureus* when exposed to green and yellow light [155]. Light intensity has also been found to influence the growth and pigment production of *M. ruber* under submerged fermentation [156]. Another study on the influence of moisture content of wood substrate on fungal pigment production in spalted wood was described. Based on the results, low moisture content stimulates the pigmentation in *T. versicolor and X. polymorpha*, while enhanced pigment production was observed at higher moisture content in the case of *I. hispidus*, *L. brumalis (*formerly known as *P. brumalis), C. squamosus (*formerly known as *P. squamosus)*, and *S. cuboideum* [34,159]. Optimization of pigment production by simultaneously altering the physical and chemical parameters has been explored by many investigators. Several studies have reported an enhancement of the yield of pigment and biomass from different fungal genera such as *Monascus*, *Penicillium*, *Fusarium*, *Alternaria*, etc., when the physical and chemical parameters were simultaneously altered [104,133,135,158,160,161,162,163,164,165,166,167].

Nowadays, co-culturing has been found to be an effective method for the activation of cryptic pathways via cell–cell interactions, which ultimately results in the production of novel secondary metabolites such as pigments from the fungi [168,169]. Studies have reported that the induction or enhancement in pigment production was possible using co-culturing of fungi with bacteria or yeast, but it was species-specific. In case of *Monascus* and *A. chevalieri*, co-culturing was found to be effective, whereas in case of *F. oxysporum*, the results were negative [158,170]. Co-culturing of *C. neoformans* with *Klebsiella aerogenes* led to synthesis of melanin by the fungus, using dopamine synthesized by bacteria [171]. Researchers have also found that many fungi produce different types of zone lines when co-cultured with other fungi. Zone lines are narrow, dark marks composed of pigments (primarily melanin) produced in decaying wood by fungi in response to other fungi, to self-isolate from other decaying fungi and protect their resources [172]. It has been observed that many white rot fungi such as *T. versicolor*, *Stereum gausapatum*, *Bjerkandera adusta*, *X. polymorpha*, and few brown rot fungi (*Poria weirii*, *Piptoporus betulinus*) produce zone lines upon detection of another fungus in their territory [173]. *T. versicolor* and *B. adusta* were found to be the best fungal pair which produce zone lines upon co-culturing, whereas *X. polymorpha* produces zone lines individually in the absence of other fungi [174]. This clearly reveals that the method of co-culturing of these fungi has a significant impact on their pigment production which supplies pigments used for coloring different types of woods in order to enhance their market value.

Various modes of cultivation and various methods and techniques of pigment extraction were investigated by several researchers to enhance fungal pigment production and recovery. Different strategies such as the use of different surfactants (Tween 80, Span 20, Triton X-100, and polyethylene glycerol polymer 8000), different solvents (acetone, acetonitrile, chloroform, cyclohexane, chloramphenicol, dichloromethane, dimethyl sulfoxide, hexane, isooctane, methanol, methyl sulfoxide, pyridine, tetrahydrofuran, and water), and potential extraction techniques (pressurized liquid extraction technique) have also been assessed, compared, and confirmed by researchers for the rapid extraction and enhanced recovery of pigments from submerged fermentation [72,134,175,176,177]. Researchers also suggested the use of shake culture methods using water as a carrier instead of using wood-based malt–agar media for pigment production from wood-degrading fungi [178].

Genetic engineering techniques for enhanced pigment production in fungi have been reported [1,20,179]. Certain genetic approaches such as alteration or modifications of genes, cloning of genes, or elimination of non-essentilal genes (mycotoxins) have been investigated for increasing pigment production and reducing mycotoxins production in fungi [180,181,182]. The manipulation of biosynthetic pathways has also been investigated by researchers for boosting fungal pigment production. A study on *F. graminearum* has shown that the transcription factor AurR1 has a positive regulatory effect on the aurofusarin gene cluster, enhancing the production of aurofusarin [183]. A recent study on *Monascus* strains, revealed that transcription factors play an important regulatory role in pigment diversity [184]. More research on this aspect may lead to enhanced pigment production.

## 5. Applications or Biological Activities of Fungal Pigments

Many fungal pigments have been reported to have a variety of biological applications because of their different properties such as antimicrobial, antioxidant, anticancer, and cytotoxic activities in addition to coloring property [1,20,25,179]; however, the degree of purity of pigments investigated in the various studies is not always known. 

### 5.1. Fungal Pigments as Food Colorants

The majority of work done on fungal pigments is related to their use as food colorants. The possibility of the use of fungal pigments in different industries, particularly in the food industry, has been revealed long ago by many researchers [9,25,46,48,179,185,186,187]. The potential of fungal pigments to be used as food colorants or as food additives in different food products has been assessed by many researchers [51,188]. Some of the fungal pigments have already entered into the market as food colorants such as *Monascus* pigments, arpink red from *P. oxalicum*, riboflavin from *Ashbya gossypii*, and β-carotene from *B. trispora* [12,25,189].

### 5.2. Fungal Pigments as Antimicrobial Agents

Numerous microbial pigments have been reported to possess many health benefits over synthetic pigments [8,14]. Several studies have proved that the pigments or pigment extracts of certain species of fungal genera (*Monascus*, *Fusarium*, *Talaromyces*, *Trichoderma*, *Penicillium*, and *Aspergillus*) and yeast *R. glutinis* possess antimicrobial activity against different pathogenic bacteria as well as yeast and fungi. All these studies suggest the potential use of bioactive pigments as food preservatives or as antibacterial ingredients in the food and pharmaceutical industries [19,66,70,82,135,166,189,190,191,192,193,194]. Similarly, the antimicrobial potential against selected pathogenic bacteria of different types of fabrics (cotton, silk, etc.) dyed with pigments of fungi (*A. alternata* and *Thermomyces* spp.) has also been evaluated, and positive results of these studies suggest their possible use in producing specific products for medical application, such as bandages, suture threads, face masks, etc. [195,196,197].

### 5.3. Fungal Pigments as Antioxidant Agents

It has been reported that microbial pigments such as carotenoids, violacein, and naphthoquinones have antioxidant potential. Many review articles mention the antioxidant potential of pigments from certain fungi and yeast [1,17,20,179,198,199]. Studies on assessment of the antioxidant activity of the pigments of certain fungi such as *Penicillium* (*P. miczynskii*, *P. purpureogenum*, *P. purpuroscens*, *Penicillium* sp.), *Fusarium* sp., *Thermomyces* sp., *Chaetomium* sp., *Sanghuangporus baumii*, *Stemphylium lycopersici*, and species of *Trichoderma* (*T. afroharzianum*, *Trichoderma* spp.) confirm the promising antioxidant potential and their possible applications in the healthcare industry [71,97,192,200,201].

### 5.4. Fungal Pigments as Cytotoxic Agents

The cytotoxic activity of pigments of certain fungal isolates (*F. oxysporum*, *T. verruculosus*, and *Chaetomium* spp.) has been assessed by many researchers using different methods such as sour orange seeds toxicity assay or yeast toxicity test (YTT) using *Saccharomyces cerevisiae*, brine shrimp lethality bioassay, or cell counting kit-8 (CCK-8) assay. These studies confirm the possible application of pigments in different industries, especially in health and pharmaceutical ones [47,82,106,202]. A latest study on the evaluation of dermal toxicity of pigments of *Thermomyces* spp. and *P. purpurogenum* in Wistar rats has revealed the nontoxic nature of pigments and suggested its potential application in cosmetics and dyeing [203].

### 5.5. Fungal Pigments as Anticancer Agents

**Figure 12:**
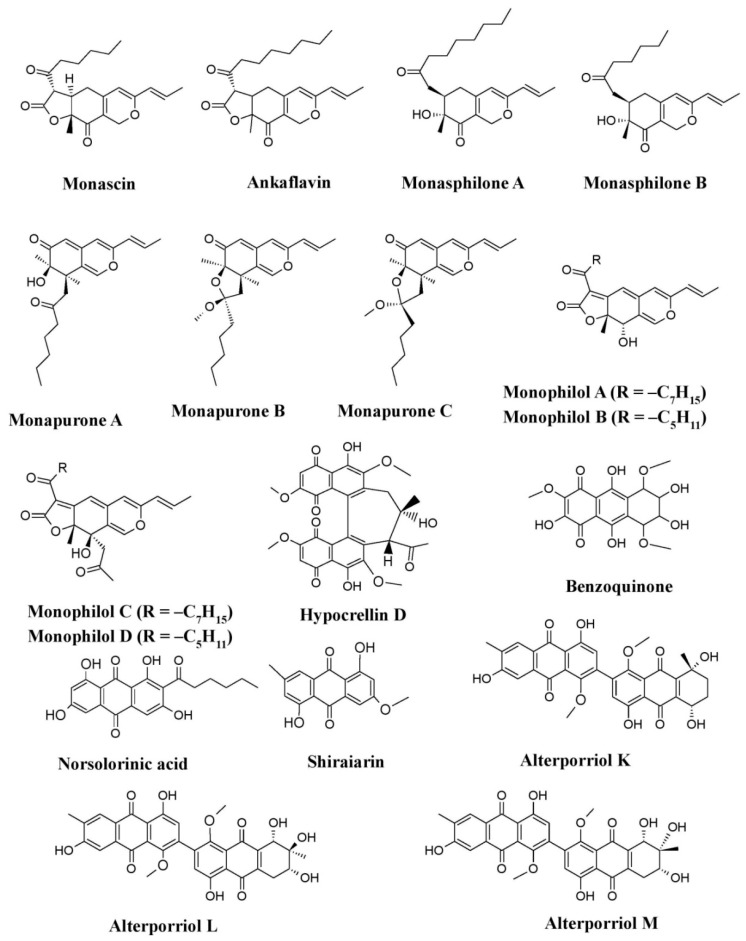
Pigments from different taxonomic groups of fungi having promising anticancer or antitumor potential, re-drawn from [32,56,57,58,62,68,88,89,113,204,205].

Fungal pigments are known to possess anticancer/antitumor activity. Several studies have revealed the fungal pigments as a potential anticancer drug. Pigments of *Monascus* species (*M. purpureus* and *M. pilosus*) such as monascin, ankaflavin, monaphilone A–B, monasphilone A–B, monapilol A–D, and monapurone A–C have been proved to possess anticancer/antitumor potential against different types of cancers, such as mouse skin carcinoma, human laryngeal carcinoma, human colon adenocarcinoma, human hepatocellular carcinoma, and pulmonary adenocarcinoma (Figure 12) [32,56,57,58,204,205]. Besides *Monascus*, pigments from other fungi such as norsolorinic acid from *A. nidulans*, shiraiarin from *Shiraia bambusicola*, alterporriol K, alterporriol L, and alterporriol M from *Alternaria* spp., benzoquinone from *Fusarium* spp., and an uncharacterized red pigment from *F. chlamydosporum* have also been reported to have anticancer, antitumor, or antiproliferative activity mainly against human breast cancer cell lines (MCF-7, MDA-MB-435, and MCF-7 b), whereas hypocrellin D from *S. bambusicola* shows anticancer activity against other cancer cell lines (Bel-7721, A-549, and Anip-973) (Figure 12) [62,68,88,89,113].

### 5.6. Fungal Pigments in the Cosmetic Industry

As the demand for natural products is increasing in the market, cosmetic industries are also in search of new types of natural pigments to replace synthetic pigments. Among the natural pigments, the use of fungal pigments is also rapidly expanding in cosmetics because of their advantages. Fungal pigments, especially melanin, carotenoids, lycopene, etc., have been reported for their application in cosmetics, sunscreens, sun lotions, sunblocks, face creams, anti-aging facials, etc. [1,206,207]. Excitingly, some of the fungal pigments (*Monascus* pigments and *Monascus*-like pigments) have already entered the market for their application in cosmetics such as skin conditioning and skin care products, lipsticks, etc. [25].

### 5.7. Fungal Pigments in the Textile Industry

The textile industry is the largest industry after agriculture in terms of economic contribution and employment generation. It majorly depends on synthetic dyes for dyeing different types of fabrics (cotton, silk, and wool). Currently, natural pigments from fungi, with their many advantages (eco-friendly, non-toxic, easy degradation, high colorfastness, high staining capability, etc.) over hazardous synthetic pigments, have proven to be a good alternative to the synthetic dyes in the textile industry. Many investigations have shown that organic pigments produced by fungi have extensive applications in the textile industry [1,5,8,18,25,207]. 

The literature reveals that only a handful of studies have investigated the application of fungal pigments in the textile industry, especially for dyeing different types of fabrics, such as cotton, silk, and wool. Various studies on the dyeing potential of pigments of different species of fungal genera (*Monascus*, *Fusarium*, *Aspergillus*, *Penicillium*, *Talaromyces*, *Trichoderma*, *Alternaria*, *Curvularia*, *Chlorociboria*, *Scytalidium*, *Cordyceps*, *Acrostalagmus*, *Bisporomyces*, *Cunninghamella*, *Thermomyces,* and *Phymatotrichum*) for different types of fabrics such as wool, cotton yarn, silk, polyester, and nylon have been reported [37,42,47,106,108,124,195,196,208,209,210,211]. Studies on the dyeing potential of pigments from wood spalting fungi (red pigment from *S. cuboideum*, yellow pigment from *S. ganodermophthorum*, and green pigment *C. aeruginosa*) have shown the possible use of these pigments for deying bleached cotton, spun polyacrylic, spun polyamide (nylon 6.6), worsted wool, spun polyester (Dacron 54), and garment fabrics, because of their high stability and good colorfastness to washing [37,212]. Another study has revealed that natural oils cannot be used in conjunction with these fungal pigments, as these fungal pigments are unstable in natural oils [42]. Results of all these studies have shown that these fungal pigments have good color stability, colorfastness properties, and dye uptake potential. Moreover, these fungal pigments do not have any adverse effects on fabric and are non-toxic to human skin. Therefore, the scope of applications of fungal pigments has the opportunity to expand into the textile and clothing industry.

### 5.8. Fungal Pigments in Dyeing Woods or as Color Modifiers

Pigment produced by wood-decaying fungi such as *T. versicolor*, *X. polymorpha*, *I. hispidus*, *S. cuboideum*, *B. adusta*, *C. aeruginascens*, and *Arthrographis cuboidea* have been used for dyeing different types of wood samples to increase their commercial importance [173,174,213]. Researchers have successfully used the red, green, and yellow pigments obtained from *S. cuboideum*, S*. ganodermophthorum*, and *C. aeruginosa*, respectively, to attenuate the presence of blue stain on wood samples of *Pinus* spp. [39].

### 5.9. Fungal Pigments in (Opto) Electronics

A recent study of the (opto)electronic properties of blends of the pigment xylindein extracted from *C. aeruginosa* has revelaed that this pigment has high photostability and electron mobility in amorphous films, which suggests its possible use for the development of sustainable, organic semiconductor materials [214,215].

## 6. Conclusions

Several advantages of fungal pigments over synthetic pigments have increased the demand for fungal pigments worldwide in recent years. This increased public awareness, eco-safety, and health concerns as well as the application of strict environmental and ecological rules and regulations, have challenged researchers to undertake both qualitative and quantitative research on pigments derived from clean, eco-friendly bio-resources, such as fungi, having minimal ecological negative impacts. Therefore, there is a necessity to explore other novel, safe pigments from the diverse taxonomic group of fungi, to meet the existing demand of eco-friendly pigments. Though several fungal strains are known as pigment producers, a large number of fungi have not been systematically explored for their pigment-producing capability. Therefore, there is a great need to explore the vast fungal diversity for rare, novel, safe pigments, using appropriate tools and techniques. A review of the literature revealed that most of the studies focused on the application of fungal pigments in the food and healthcare industries; however, fungal pigments need to pass toxicity tests and quality tests and receive many regulatory approvals before their final entry into the market as food colorants or as drugs. Therefore, the application of fungal pigments in these areas is quite difficult. 

Moreover, meager studies on the applicability of fungal pigments in other areas such as textiles, paints, varnishes, and daily household utensils leave immense possibilities to explore the indigenous diversity of fungi for their pigment production potential and their applications in different sectors, including the textile industry. In addition to the coloring properties, the biological properties of fungal pigments may open new avenues for their use in the production of valuable textiles for medical use. This provides an extensive area of exploration to identify natural, eco-friendly fungal pigments and develop their diverse applications to satisfy the public interest and market demand.

## Figures and Tables

**Table 1:** Updated list of pigment-producing fungi and their respective pigments [25,61].

Fungal Species	Pigments	References
***Monascus*** ** species**		
*Monascus pilosus*	Citrinin (yellow)	[61]
*Monascus purpureus*	Monascin (yellow), monascorubrin (orange), monascorubramine (red), monapurone A–C (yellow), monasphilone A and B (yellow), ankaflavin (yellow), rubropunctamine (purple-red), rubropunctatin (orange), monopilol A–D (yellow), citrinin (yellow), 9–(1–hydroxyhexyl)–3–(2–hydroxypropyl)–6a–methyl–9,9a–dihydrofuro[2,3–h] isoquinoline–6,8(2H,6aH)–dione (red), uncharacterized (red)	[56,57,58,59,60,61]
*Monascus ruber*	Monascin (yellow), monascorubramine (red), monascorubrin (orange), ankaflavin (yellow), citrinin (yellow), rubropunctamine (purple-red), rubropunctatin (orange), *N*–glucosylrubropunctamine (red), *N*–glucosylmonascorubramine (red), monarubrin (pale yellow), rubropunctin (pale yellow)	[52,54,61]
*Monascus* species	Ankaflavin (yellow) *, monascorubramine (red) *, rubropunctatin (orange) *	[25]
***Fusarium*** ** species**		
*Fusarium acuminatum*, *F. avenaceum*, *F. tricinctum*	Antibiotic Y (yellow), aurofusarin (red)	[61]
*Fusarium chlamydosporum*	Uncharacterized (red)	[62]
*Fusarium culmorum*	Aurofusarin (red), fuscofusarin (yellow), rubrofusarin (red)	[61]
*Fusarium fujikuroi* (formerly known as *Fusarium moniliforme*/ *Fusarium verticillioides*)	Bikaverin (red), norbikaverin (red), *O*–demethylanhydrofusarubin (red), 8–*O*–methybostrycoidin, 2–(4–((3E,5E)–14–aminotetradeca–3,5–dienyloxy)butyl)–1,2,3,4–tetrahydroisoquinolin–4–ol (ATDBTHIQN) (pink), neurosporaxanthin (orange), β–carotene (red-orange), fusarubin (red), *O*–demethylfusarubin, *O*–methyljavanicin, *O*–methylsolaniol (orange-red)	[43,61,63,64,65]
*Fusarium graminearum*	Aurofusarin (red,) rubrofusarin (red), 5–deoxybostrycoidin anthrone (green), 6–*O*–demethyl– 5–deoxybostrycoidin anthrone (blue), purpurfusarin (purple), 6–*O*–demethyl–5–deoxybostrycoidin (yellow), 5–deoxybostrycoidin (red)	[64,66]
*Fusarium oxysporum*	2,7–dimethoxy–6–(acetoxyethyl)juglone (yellow), bikaverin (red), bostrycoidin (red), nectriafurone (yellow), norjavanicin (red), *O*–methyl–6– hydroxynorjavanicin (yellow), *O*–methylanhydrofusarubin (orange-red), *O*–methylfusarubin (red), *O*–methyljavanicin, 2–acetyl–3,8–dihydroxy–6–methoxy anthraquinone (yellow), 2–(1–hydroxyethyl)–3,8–dihydroxy–6–methoxy anthraquinone (orange), neurosporaxanthin (orange), β–carotene (red-orange), uncharacterized naphthaquinones (purple)	[43,47,61,64,67]
*Fusarium poae*, *F. sambucinum*	Aurofusarin (red)	[61]
*Fusarium solani*	Fusarubin (red), *O*–methyldihydrofusarubin (red), *O*–ethylfusarubin (red), isomarticins (red)	
*Fusarium sporotrichioides*	Aurofusarin (red), β–carotene (yellow-orange) **, lycopene (red) **	[25,61]
*Fusarium stilboides*	Antibiotic Y (yellow), aurofusarin (red), nectriafurone (yellow)	[61]
*Fusarium venenatum*	Aurofusarin (red), rubrofusarin (red)	
*Fusarium* sp.	Benzoquinone (yellow)	[68]
*Fusarium* sp. PSU–F14 and PSU–F135	Fusarnaphthoquinones B (red), fusarnaphthoquinones C (red)	[69]
***Fusicolla*** ***aquaeductuum*** ** (Formerly Known as *Fusarium aquaeductuum*)**		
*Fusicolla* * aquaeductuum*	Neurosporaxanthin (orange), β–carotene (red-orange)	[43]
***Albonectria*** *** rigidiuscula*** ** (Formerly Known as *Fusarium decemcellulare*)**		
*Albonectria rigidiuscula*	Javanicin (red–orange), fusarubin (red), anhydrojavanicin, anhydrofusarubin, bostricoidin (red), novarubin	[64]
***Trichoderma*** ** species**		
*Trichoderma harzianum*	Pachybasin (yellow), chrysophanol (orange-red), emodin (yellow), 1–hydroxy–3–methyl–anthraquinone, 1,8–dihydroxy–3–methyl–anthraquinone, T22 azaphilone	[25]
*Trichoderma polysporum*	Pachybasin (yellow), chrysophanol (orange-red), emodin (yellow)	
*Trichoderma viride*	Pachybasin (yellow), chrysophanol (orange-red), emodin (yellow), 1,3,6,8–tetrahydroxyanthraquinone, 2,4,5,7– tetrahydroxyanthraquinone	
*Trichoderma aureoviride*	Pachybasin (yellow), chrysophanol (orange-red)	
*Trichoderma afrharzianum*, *Trichoderma pyramidale*, *Trichoderma parareesei* (formerly known as *Trichoderma atroviride*), *Trichoderma* sp. 1	Uncharacterized (yellow)	[70,71]
*Trichoderma parceramosum*	Uncharacterized (red)	[72]
***Cordyceps farinosa*** ** (Formerly Known as *Isaria farinosa*)**		
*Cordyceps farinosa*	Anthraquinone derivative	[73]
***Ophiocordyceps*** ***unilateralis* (Formerly Known as *Cordyceps unilateralis*)**		
*Ophiocordyceps unilateralis*	Erythrostominone (red), 3,5,8–TMON * (red), deoxyerythrostominone (red), deoxyerythrostominol (red), 4–*O*–methyl erythrostominone (red), epierythrostominol (red), naphthoquinones (deep blood red) **	[25]
***Beauveria*** ** species**		
*Beauveria basiana*	Tenellin (yellow), bassianin (yellow), pyridovericin (pale yellow), pyridomacrolidin (pale yellow), oosporein (red)	[25,74]
*Beauveria brongniartii* (formerly known as *Beauveria tenella*)	Tenellin (yellow), bassianin (yellow)	
***Torrubiella*** ** species**		
*Torrubiella* sp.	Torrubiellones A–D (yellow)	[75]
***Lecanicillium*** ** species**		
*Lecanicillium aphanocladii*	Oosporein (red)	[41]
***Hyperdermium*** ** species**		
*Hyperdermium bertonii*	Skyrin (orange-red)	[25]
***Daldinia*** ** species**		
*Daldinia bambusicol*, *Daldinia caldariorum*, *Daldinia childiae*, *Daldinia clavata*, *Daldinia fissa*, *Daldinia grandis*, *Daldinia lloydi*, *Daldinia loculata*, *Daldinia petriniae*, *Daldinia singularis*	BNT (1,1ˊ–Binaphthalene–4,4ˊ–5,5́–tetrol) (yellow), daldinol (dark brown), 8–methoxy–1–napthol, 2–hydroxy–5–methylchromone	[25]
*Da* *ldinia concentrica*	BNT (1,1ˊ–Binaphthalene–4,4ˊ–5,5́–tetrol) (yellow), daldinol, 8–methoxy–1–napthol, 2–hydroxy–5–methylchromone, daldinal A–C (yellow), daldinin A–C (green-olivaceous-isabelline)	
*Da* *ldinia eschscholzii*	BNT (1,1ˊ–Binaphthalene–4,4ˊ–5,5́–tetrol) (yellow), daldiol (dark brown), 8–methoxy–1–napthol, 2–hydroxy–5–methylchromone, daldinal A–C (yellow)	
***Jackrogersella cohaerens*** ** (Formerly Known as *Annulohypoxylon cohaerens*)**		
*Jackrogersella cohaerens*	Cohaerin A	[25]
***Hypoxylon*** ** species**		
*Hypoxylon fragiforme*	Hypoxyxylerone (green), fragiformins A–B, cytochalasin H (white), mitorubrin azaphilones (red)	[25]
*Hypoxylon howeanum*	Mitorubrin azaphilones (red)	
*Hypoxylon lechatii*	Vermelhotin (orange-red), hypoxyvermelhotins A–C (orange-red)	
*Hypoxylon fuscum*	Daldinin A–C (green-olivaceous-isabelline)	
*Hypoxylon fulvo–sulphureum*	Mitorubrinol derivatives	
*Hypoxylon sclerophaeum*	Hypoxylone (orange)	
*Hypoxylon rickii*	Rickenyl B (red), rickenyl D (brown)	
*Hypoxylon lenormandii*, *Hypoxylon jaklitschii*	Lenormandins A–G (yellow)	
*Hypoxylon rubiginosum*	Mitorubrin (orange), rubiginosin (orange-brown), hypomiltin (yellowish-green)	
***Alternaria*** ** species**		
*Alternaria alternata*	Alternariol (red), altenuene (red-violet), alternarienoic acid (red), alternariol-5-methyl ether (red-brown), tenuazoic acid (orange-red), alterperylenol (red), stemphyperylenol (yellow–orange-red)	[76]
*Aternaria dauci*	Uncharacterized (red)	[25,61]
*Aternaria porri*	Altersolanol A (yellow-orange), dactylariol	[25,61,77]
*Aternaria solani*, *Aternaria tomatophila*	Altersolanol A (yellow-orange)	[25,61]
*Alternaria* species	Alterperylenol (red), dihydroalterperylenol (dark purple)	[78]
*Alternaria* sp.* ZJ9–6B*	Alterporriol K–M (red)	[79]
***Curvularia*** ** species**		
*Curvularia lunata*	Chrysophanol (red), cynodontin (bronze), helminthosporin (maroon), erythroglaucin (red), catenarin (red)	[25,61]
***Sanghuangporus*** **species**		
*Sanghuangporus baumii*	Uncharacterized (yellow)	[71]
***Clonostachys*** ** species**		
*Clonostachys intermedia*	Uncharacterized (yellow)	[71]
***Pyrenophora*** ** species (Previously Known as species of *Drechslera*)**		
*Pyrenophora teres*, *Pyrenophora graminea*, *Pyrenophora tritici–repentis*, *Pyrenophora grahamii*, *Pyrenophora dictyoides*, *Pyrenophora chaetomioides*	Catenarin (red), cynodontin (bronze), helminthosporin (maroon), tritisporin (reddish-brown), erythroglaucin (red)	[25,61]
***Exophiala*** **species**		
*Exophiala dermatitidis (*formerly known as* Wangiella dermatitidis)*	Melanin (black-brown)	[44]
***Sporothrix*** **species**		
*Sporothrix schenckii*	Melanin (black-brown)	[44]
***Cryptococcus*** ** species**		
*Cryptococcus neoformans*	Dihydroxy phenyl alanine-melanin	[29,80]
***Tuber*** **species**		
*Tuber melanosporum*	Melanin (black)	[29,81]
***Polyporus*** ** species**		
*Lentinus brumalis* (formerly known as *Polyporus brumalis*)	Melanin (black)	[34,35]
*Cerioporus squamosus* (formerly known as *Polyporus squamosus*)	Melanin (black)	
***Xylaria*** ** species**		
*Xylaria polymorpha*	Melanin (black)	[34,35]
***Fomes*** **species**		
*Fomes fomentarius*	Melanin (black)	[34,35]
***Oxyporus*** **species**		
*Oxyporus populinus*	Melanin (black)	[34]
***Trametes*** ** species**		
*Trametes versicolor*	Melanin (black)	[34,35]
***Inonotus*** ** species**		
*Inonotus hispidus*	Melanin (black), uncharacterized (yellow)	[34,35,36]
***Chlorociboria*** ** species**		
*Chlorociboria aeruginascens*	Xylindein (green), xylindein quinol (yellow)	[33]
*Chlorociboria aeruginosa*	Xylindein (green)	[37,39]
***Scytalidium*** ** species**		
*Scytalidium cuboideum*	Draconin red (red)	[37,39]
*Scytalidium ganodermophthorum*	Uncharacterized (yellow)	[36,39]
*Scytalidium lignicola*	Uncharacterized (yellow)	[36,39]
***Epicoccum*** ** species**		
*Epicoccum nigrum*	Carotenoids, chromanone (yellow), epicoccarines A–B, epicocconone (fluorescent yellow), epipyridone (red), flavipin (brown), isobenzofuran derivatives (yellow to brown), orevactaene (yellow)	[41,61]
***Chaetomium*** ** species**		
*Chaetomium cupreum*	Oosporein (red), rotiorinols A–C (red), rubrorotiorin (red)	[25]
*Chaetomium globosum*	Chaetoviridins A–D (yellow), chaetoglobin A–B, chaetomugilins A–F, cochliodinol (purple)	
*Chaetomium* sp. NA–S01–R1	Chaephilone–C (yellow), chaetoviridides A–C (red)	[82]
***Achaetomium*** ** species**		
*Achaetomium* sp.	Parietin (orange)	[25]
***Phyllosticta*** ** species**		
*Phyllosticta capitalensis*	Melanin (black)	[83]
***Cladosporium*** ** species**		
*Cladosporium cladosporioides*	Calphostins A–D and I (red)	[61]
***Nodulisporium* species**		
*Nodulisporium hinnuleum*	Hinnuliquinone (red)	[84]
***Astrosphaeriella* species**		
*Astrosphaeriella papuana*	Astropaquinones A–C (orange)	[85]
***Arthrobotrys* species**		
*Arthrobotrys ferox*	Carotenoid	[86]
***Thelebolus* species**		
*Thelebolus microsporus*	β-carotene (orange)	[86,87]
***Shiraia* species**		
*Shiraia bambusicola*	Shiraiarin (red), hypocrellin D (orange-red)	[88,89]
***Paecilomyces*** ** species**		
*Paecilomyces sinclairii*	Uncharacterized (red) **	[25,61]
***Neurospora*** ** species**		
*Neurospora crassa*	Neurosporaxanthin (yellow-orange), phytoene (yellow-orange), β–carotene (red-orange), lycopene (red), neurosporen (yellow-orange), spirilloxanthin (violet), ϒ–carotene (yellow-orange), β–carotene (yellow-orange) **	[25,90]
*Neurospora sitophila*	Neurosporaxanthin (yellow-orange)	[26]
*Neurospora intermedia*	Uncharacterized (yellow-orange), a mixture of carotenoids	
***Blakeslea*** **species**		
*Blakeslea trispora*	β–carotene (yellow-orange) *, lycopene (red) *	[25]
***Ashbya* species**		
*Ashbya gossypi*	Riboflavin (yellow) *	[25]
***Phycomyces* species**		
*Phycomyces blakesleeanus*	β–carotene (yellow-orange) **	[25]
***Mucor* species**		
*Mucor circinelloides*	β–carotene (yellow-orange) ***	[25]
***Lactarius* species**		
*Lactarius* sp.	Azulenes (blue) **	[25]
***Penicillium*** ** species**		
*Penicillium atramentosum*	Uncharacterized (dark brown)	[61,91]
*Penicillium atrosanguineum*	Phoenicin (red), uncharacterized (yellow and red)	
*Penicillium atrovenetum*	Atrovenetin (yellow), norherqueinone (red)	
*Penicillium aurantiogriseum*	Uncharacterized	
*Penicillium brevicompactum*, *Penicillium simplicissimum*	Xanthoepocin (yellow)	
*Penicillium chrysogenum*	Sorbicillins (yellow), xanthocillin (yellow), chrysogine (yellow)	[61,92]
*Penicillium citrinum*	Anthraquinones (yellow), citrinin (yellow)	[61]
*Penicillium convolutum* (formerly known as *Talaromyces convolutus*)	Talaroconvolutins A–D, ZG–1494α	[93]
*Penicillium cyclopium*	Viomellein (reddish–brown), xanthomegnin (orange)	[61]
*Penicillium discolor*	Uncharacterized	
*Penicillium echinulatum*	Uncharacterized (yellow)	
*Penicillium flavigenum*	Xanthocillin (yellow), dihydrotrichodimerol (yellow)	[41,61]
*Penicillium freii*, *Penicillium viridicatum*	Viomellein (reddish-brown), vioxanthin, xanthomegnin (orange)	[61]
*Penicillium herquei*	Atrovenetin (yellow), herqueinones (red and yellow)	
*Penicillium melinii*	Atrovenetin (yellow)	[91]
*Penicillium miczynskii*	Uncharacterized (red)	[71]
*Penicillium mallochii*	Sclerotiorin (yellow)	[94]
*Penicillium oxalicum*	Arpink red™, anthraquinone derivative (red), secalonic acid D (yellow), anthraquinones (red and other hues) *	[25,61]
*Penicillium paneum*	Uncharacterized (red)	[61]
*Penicillium persicinum*	Uncharacterized (cherry red)	
*Penicillium* sp. AZ	PP–V (violet), PP–R (red)	[95]
*Penicillium* sp. (GBPI_P155)	Uncharacterized (orange)	[96]
*Penicillium* sp. NIOM–02	Uncharacterized (red)	[97]
*Penicillium* sp.	Uncharacterized (red)	[98,99]
***Talaromyces*** ** species**		
*Talaromyces aculeatus* (formerly known as *Penicillium aculeatum*)	Uncharacterized	[61]
*Talaromyces atroroseus*	Mitorubrin (red), monascorubrin (red), PP–R (red), glauconic acid (red), purpuride (red), ZG–1494α (red), azaphilones (red) ***	[25,100]
*Talaromyces albobiverticillius*, *Talaromyces amestolkiae*, *Talaromyces stollii*	*Monascus*–like azaphilones (red)	[25]
*Talaromyces cnidii*, *Talaromyces coalescens*	*Monascus*–like azaphilones (red), uncharacterized (red)	
*Talaromyces funiculosus* (formerly known as* Penicillium funiculosum*)	Ankaflavain (yellow), uncharacterized	[61]
*Talaromyces islandicus* (formerly known as* Penicillium islandicum*)	Emodin (yellow), skyrin (orange), erythroskyrin (orange-red), luteoskyrin (yellow)	
*Talaromyces marneffei* (formerly known as* Penicillium* *marneffiei*)	Monascorubramine (purple-red), mitorubrinol (orange-red), rubropunctatin (orange), purpactin, herqueinone like (brick red), secalonic acid D (yellow)	[61,101]
*Talaromyces pinophilus* (formerly known as* Penicillium pinophilum*)	Azaphilones, uncharacterized	[25,61]
*Talaromyces purpureogenus* (formerly known as *Penicillium purpureogenum)*	Mitorubrin (yellow), mitorubrinol (orange-red), PP–R (purple-red), purpurogenone (yellow-orange), rubropunctatin (red), *N*–glutarylmonascorubramine, *N*–glutarylrubropunctamine, uncharacterized (red), azaphilones (red) ***	[25,61,102,103,104,105]
*Talaromyces ruber* (formerly known as* Penicillium crateriforme*)	Uncharacterized, *Monascus*–like azaphilones	[25]
*Talaromyces rugulosus* (formerly known as* Penicillium rugulosum*)	Rugulosin (yellow)	[61]
*Talaromyces variabillis* (formerly known as *Penicillium variabile*)	Rugulosin (yellow)	[61]
*T* *alaromyces* * vericulosus*	Uncharacterized (red)	[106]
*Talaromyces* sp. DgCr22.1b	Talaroxanthone (yellow)	[107]
*Talaromyces siamensis*, *Talaromyces* sp.	Uncharacterized (red)	[71,108]
*Talaromyces* sp.	*N*–threonine rubropunctamine (red)	[72]
***Hamigera avellanea*** ** (Formerly Known as *Talaromyces avellaneus*)**		
*Hamigera avellanea*	Emodin (yellow), erythroglaucin (red), catenarin (red)	[109]
***Aspergillus*** ** species**		
*Aspergillus amstelodami*	Physcion (yellow), erythroglaucin (red), flavoglaucin (yellow), auroglaucin (orange-red)	[25]
*Aspergillus awamori*	Asperenone (yellow)	[110]
*Aspergillus chevalieri*	Physcion (yellow), erythroglaucin (red), flavoglaucin (yellow), auroglaucin (orange-red), catenarin (red), rubrocristin (red)	[25]
*Aspergillus cristatus*	Emodin (yellow), questin (yellow to orange-brown), erythroglaucin (red), physcion (yellow), catenarin (red), rubrocristin (red)	[25,61]
*Aspergillus echinulatum*, *Aspergillus glaber*, *Aspergillus spiculosus*, *Aspergillus umbrosus*	Erythroglaucin (red), physcion (yellow), catenarin (red), rubrocristin (red)	[25]
*Aspergillus fumigatus*	Melanin (dark brown-black)	[25,111]
*Aspergillus falconensis*, *Aspergillus fruticulosus*	Falconensins A–H (yellow), falconensones A1 and B2 (yellow), zeorin (yellow)	[25]
*Aspergillus glaucus*	Physcion (yellow), emodin (yellow), questin (yellow to orange-brown), erythroglaucin (red), catenarin (red), rubrocristin (red), flavoglaucin (yellow), auroglaucin (orange-red), aspergin (yellow)	[25,61]
*Aspergillus intermedius*, *Aspergillus leucocarpus*, *Aspergillus tonophilus*	Physcion (yellow), erythroglaucin (red)	
*Aspergillus ochraceus*	Viomellein (reddish-brown), vioxanthin, xanthomegnin (orange)	
*Aspergillus melleus*, *Aspergillus sulphureus*, *Aspergillus westerdijkiae*	Viomellein (reddish-brown), rubrosulphin (red), viopurpurin (purple), xanthomegnin (orange)	
*Aspergillus nidulans*	Ascoquinone A (red), norsolorinic acid, sterigmatocystin (yellow), melanin (dark brown-black)	[25,112,113]
*A* *spergillus niger*	Flavioline (orange-red), *N*-naptho–γ–pyrones (yellow), aspergillin (black), azanigerones A–F, asperenone (yellow), melanin (dark brown-black)	[25,61,110,114,115]
*A* *spergillus nishimurae*	Anishidiol (yellow)	[116]
*Aspergillus parvathecia*, *Aspergillus rugulosus*, *Aspergillus versicolor*	Sterigmatocystin (yellow)	[25]
*A* *spergillus* * purpureus*	Epurpurins A–C (yellow)	
*A* *spergillus* * repens*	Emodin (yellow), physcion (yellow), erythroglaucin (red), catenarin (red), rubrocristin (red), questin (yellow to orange-brown)	
*A* *spergillus* * ruber*	Catenarin (red), rubrocristin (red), emodin (orange), asperflavin (yellow), eurorubrin (Brown), questin (yellow to orange-brown), 3–*O*–(α–D–ribofuranosyl)–questin (orange), 2–*O*–methyl–9–dehydroxyeurotinone, 2–*O*–methyl–4–*O*–(α–D–ribofuranosyl)–9–dehydroxyeurotinone, 2–*O*–methyleurotinone	[25,117]
*A* *spergillus sclerotioniger*	Uncharacterized (yellow)	[61]
*A* *spergillus sclerotiorum*	Neoaspergillic acid (yellow-green)	[91]
*A* *spergillus* * terreus*	Uncharacterized (yellow)	[118]
*Aspergillus* sp.	Ferriaspergillin (red), ferrineoaspergillin (red)	[119]
*Aspergillus* sp.	Uncharacterized (yellow)	[120]

* Industrial production (IP), ** research project (RP), *** development stage (DS).
